# Genome-Wide Identification of Seven Polyamine Oxidase Genes in *Camellia sinensis* (L.) and Their Expression Patterns Under Various Abiotic Stresses

**DOI:** 10.3389/fpls.2020.544933

**Published:** 2020-09-04

**Authors:** Mengshuang Li, Jing Lu, Mingmin Tao, Mengru Li, Hua Yang, En-hua Xia, Qi Chen, Xiaochun Wan

**Affiliations:** ^1^State Key Laboratory of Tea Plant Biology and Utilization, Anhui Agricultural University, Hefei, China; ^2^College of Science, Anhui Agricultural University, Hefei, China; ^3^Key Laboratory of Food Nutrition and Safety, School of Tea and Food Science & Technology, Anhui Agricultural University, Hefei, China

**Keywords:** polyamine oxidase, polyamines, *Camellia sinensis*, stress response, genomic characterization

## Abstract

Polyamines (PAs) in plant play a critical role in growth and development and in response to environmental stress. Polyamine oxidase (PAO) is a flavin adenine dinucleotide dependent enzyme that plays a major role in PA catabolism. For the first time, *PAO* genes in tea plant were screened for the whole genome-wide and seven *CsPAO* genes were identified, which were named *CsPAO1-7*. Phylogenetic tree analysis revealed seven CsPAO protein sequences classed into three groups, including clade I, III, and IV. Compared with other plants, the tea plant lacked clade II members. Genetic structure and tissue specific expression analysis showed that there were significant differences among members of the *CsPAO* gene family. Among members of the *CsPAOs* family, *CsPAO4* and *CsPAO5* contain more introns and are highly expressed in various organizations. *CsPAO1*, *CsPAO4*, and *CsPAO5* genes were cloned and expressed heterologously to verify theirs function. Heat map showed high response of *CsPAO5* to drought stress, while *CsPAO*1 and *CsPAO*2 were sensitive to changes in nitrogen nutrition. Furthermore, exogenous abscisic acid (ABA) treatment indicated that the expression of most *CsPAO* genes in roots and leaves was significantly induced. In the root, Spm content increased significantly, while Put and Spd content decreased, suggesting that ABA has great influence on the biosynthesis of PAs. Anaerobic treatment of picked tea leaves showed that the decomposition of PAs was promoted to a certain extent. The above data help to clarify the role of *CsPAO* in response abiotic and nitrogen nutritional stresses in tea plants, and provide a reference perspective for the potential influence of PAs on the tea processing quality.

## Introduction

Polyamines (PAs) are aliphatic nitrogen-containing bases, with low molecular weight and biological activity. Putrescine (Put), spermidine (Spd), and spermine (Spm) are the three most common PAs found in plants (Knott et al., 2007; Takano et al., 2012). PAs have many important functions in plants. They play a vital role in flowering and development (Ahmed et al., 2017), leaf development and senescence (Sobieszczuk-Nowicka et al., 2015; Sobieszczuk-Nowicka, 2017), and fruit development and maturity (Liu and Moriguchi, 2007; Fortes and Agudelo-Romero, 2018). In addition, PAs are key components of plant abiotic stress responses (Liu et al., 2015; Montilla-Bascon et al., 2017), and their response mechanisms include participation in protein regulation (Sayas et al., 2019), regulation of chemical penetration and photoprotection of chloroplasts (Ioannidis et al., 2016), and control of N/C balance in plants (Moschou et al., 2012). Furthermore, many studies have shown that the application of exogenous PAs enhanced the tolerance of plants to several abiotic stresses such as saline, drought, water logging, osmosis, heavy metals, and extreme temperatures (Alcazar et al., 2006; Handa and Mattoo, 2010; Ogawa and Mitsuya, 2011; Nambeesan et al., 2012; Pál et al., 2019; Yin et al., 2019). The above studies indicated that the intracellular PA level is regulated by the dynamic balance of biosynthesis and catabolism, which is crucial for maintaining the normal growth and development of plants.

Flavin-containing polyamine oxidase (PAO) is involved in PA catabolism in plants (Tavladoraki et al., 2016). PAOs can be divided into two categories according to their involvement in the process of PA catabolism, one is called the terminal catabolism (TC) pathway and the other is called the back-conversion (BC) pathway (Moschou et al., 2008; Angelini et al., 2010; Kusano et al., 2015). In the TC pathway, PAOs oxidize Spd and Spm to produce H_2_O_2_, 1, 3-diaminopropane (DAP), 4-aminobutyraldehyde (Spd decomposition), or N-(3-aminopropyl)-4-aminobutyraldehyde (Spm decomposition). Maize *PAO* (*ZmPAO*) was the earliest identified *PAO* family member (Tavladoraki et al., 1998), followed by barley *PAO* (*HvPAO1* and *HvPAO2*), rice *PAO* (*OsPAO7*), and sweet orange *PAO* (*CsPAO4*), which were all proven to catalyze the TC pathway of PAs (Cervelli et al., 2001; Liu et al., 2014c; Wang and Liu, 2016). In the BC pathway, PAO converted Spm to Spd, and Spd to Put, then the reverse reaction of PA synthesis usually generated 3-aminopropanal and H_2_O_2_ (Moschou et al., 2012; Tavladoraki et al., 2016). All the five *PAOs* (*AtPAO1-AtPAO5*) in *Arabidopsis* have been reported to catalyze the BC reaction of PAs (Tavladoraki et al., 2006; Moschou et al., 2008; Kamada-Nobusada et al., 2008; Fincato et al., 2010; Zarza et al., 2017). There were four *PAOs* (*OsPAO1, OsPAO3, OsPAO4, and OsPAO5*) in rice (*OsPAO1-7)* that performed the BC reaction (Ono et al., 2012; Liu et al., 2014a; Liu et al., 2014b). Similarly, four of the seven *PAOs* identified in tomato (*SlPAO1-SlPAO7*) were thought to perform PA conversion reaction (Hao et al., 2018).

In order to further understand the characteristics of PA metabolism in tea plant, we screened the whole genome of tea plant, identified seven *PAO* gene sequences, and named them *CsPAO1-7*. Three *PAOs* (*CsPAO1*, *CsPAO4*, and *CsPAO5*) were selected for gene cloning and heterologous expression to verify their functions. Subsequently, the transcriptome data in NCBI combined with qRT-PCR results were used to analyze the tissue-specific expression of the *CsPAO* genes and their response to different abiotic stresses. Moreover, the changes of PA contents were detected to analyze the respective roles of the *CsPAOs*.

## Materials and Methods

### Identification and Biological Analysis of CsPAOs

To screen the PAO gene in the *Camellia sinensis* genome, the reported *AtPAO* sequences were downloaded from the *Arabidopsis* gene annotation database TAIR (http://www.arabidopsis.org/), the reported *OsPAO* sequences were obtained from the *Oryza sativa* genome annotation database RCAR (http://Oryza.sativa.plantbiology.msu.edu/), and the *PAO* sequences of other species were obtained from NCBI. All of these genes were used as a basis for blast searches in the *Camellia sinensis* genome annotation database of TPIA (http://tpia.teaplant.org/) (Xia et al., 2019) to obtain the homologous *CsPAO* coding sequences, genomic sequences and polypeptide sequences. The molecular weight (MV) and isoelectric point (PI) of the identified polypeptides were calculated by ExPasy (http://web.expasy.org/compute_pi/) (Gasteiger et al., 2005), and the cellular localization was predicted using Wolf PSORT Prediction (http://www.wolfpsort.org,html) (Paul et al., 2007).

All *CsPAO* cDNA sequences were aligned with the corresponding genomic sequences using the Gene Structure Display Server 2.0 (http://gsds.cbi.pku.edu.cn/index.php) (Hu et al., 2014) to perform gene structure analysis. Multiple sequence alignments of the *CsPAO* genes were performed using DNAMAN. PAO sequences in 26 plants were used for a phylogenetic tree construction with MEGA7 (Kumar et al., 2016). Tissue-specific expression was examined by qRT-PCR, and plotted by ITOL (https://itol.embl.de/) (Letunic and Bork, 2019).

### Plant Materials and Abiotic Stress Treatment

The perennial tea plants [*Camellia sinensis* (L.) O. Kuntze] used in this study were grown in the experimental plantation of Anhui agriculture university at the Dayangdian in Hefei, Anhui province, China. Nine organs of Longjin 43^#^ variety were sampled, including the buds, first leaves, second leaves, third leaves, fourth leaves, tender stems, fibrous roots, flowers, and tea seeds at the right season. The samples were immediately frozen in liquid nitrogen and stored in a -80°C ultra-refrigerator until freeze-drying. These samples were used for tissue expression analysis of *CsPAOs*. Three biological replicates of each sample were taken for subsequent testing.

Two-year-old tea seedings (Longjin 43^#^) were obtained from Dechang tea plantation (Shucheng, Anhui, China) and used for the ABA treatment experiment. After 1 week of clear water feeding, Shigeki Konish nutrient solution was used for hydroponic culture, and the growth conditions were maintained at 23 ± 2°C and 70 ± 10% relative humidity. When the tea seedlings grew new fibrous roots, 0.25 mM ABA was added to the culture solution. Fibrous roots and tender leaves were collected after treatment for 0, 0.5, 2, 6, and 24 h. Samples were quickly frozen, and used for qRT-PCR to analyze the expression patterns of *CsPAO* genes and LC-MS to detect the content of PAs.

Young shoots (one bud with two leaves) of clonal tea variety (Longjin 43^#^) were picked in spring tea season, and divided into three portions for the processes of natural spreading, filled-N_2_, and filled-CO_2_, then the samples were collected at 0, 1, and 6 h. The sample processing method was the same as above. Three biological replicates of each sample were taken for subsequent testing.

### RNA Extraction and Real-Time Quantitative qRT-PCR Analysis

Total RNA of each sample was extracted using the RNAprep pure plant kit (polysaccharide & polyphenolic rich) (TIANGEN, Shanghai, China), and reverse transcription was performed using the PrimeScriptTMRT reagent kit (TaKaRa, Dalian, China). The *CsGAPDH* gene was used as an internal reference for qRT-PCR analysis. More than two pairs of primers were designed for all selected genes in this study using Primer plus 3.0 (http://www.primer3plus.com/cgi-bin/dev/primer3plus.cgi), and only primers with a single melting curve and the amplification efficiency between 95%–105% were screened for the subsequent qRT-PCR detection. All screened gene-specific primers are listed in **Supplemental Table 1**. TaKaRa TB Green^®^ Premix Ex TaqTM II kit (TAKARA Bio, Japan) was chosen for qRT-PCR, and the instrument used was QuantStudio™ 6 Flex Real-Time PCR System (Thermo Fisher Scientific, Singapore). Every sample was tested in three biological replicates and three technical replicates, which showed good consistency. The relative expression level was transformed log2 and calculated by the 2^-ΔCt^ method (Livak and Schimittgen, 2001).

### Transcriptome Data Analysis

The responses of three gene family members to cold (Wang et al., 2013), drought and salt (Zhang et al., 2017) were obtained from NCBI, and gene expression levels were calculated as reads or fragments per kilobase of exon model per million mapped reads (RPKM/FPKM). The competitive expression (the thresholds of log2 FPKM treatment/FPKM control) was used to prepare a heatmap using the Multiple Array Viewer software (Saeed et al., 2003). The accession number of the cold-processed RNA-Seq dataset is SRA061043. The accession number for the PEG and salt-treated RNA-Seq dataset is PRJEB11522.

Transcriptome data of nitrogen nutritional stress in the *CsPAO* gene family were published by our laboratory (Yang et al., 2020). In the control group, tea seedlings were hydroponically cultured with Shigeki Konishi nutrient solution. In the treatment group, other nutrients remained unchanged except for the addition of different nitrogen sources [ammonium nitrogen: 0.715 mM (NH_4_)_2_SO_4_; nitrate nitrogen: 0.715 mM Ca(NO_3_)_2_; ethylamine: 1.43 mM ZtNH_2_.HCl] equal to that in the control group. In the nitrogen-deficient group, other components remained unchanged except for the absence of nitrogen. The data is the results of 10 days after processing.

### Cloning and Functional Verification of Partial CsPAO Genes

The full length sequences of *CsPAO1*, *CsPAO4*, and *CsPAO5* were cloned by RACE-PCR. The primers are listed in **Supplemental Table 2**. High-fidelity enzyme KOD-Plus-Neo was used for amplification (Toyobo Life Science, Japan). The target genes were transferred into the expression vector pGEX-4T-2 for protein expression, and induced by 5 mM isopropyl-beta-D-thiogalactopyranoside (IPTG) at 16°C for 24 h. The fusion protein was detected by SDS-PAGE. The PAO enzyme activity of the purified protein was detected using a special kit (Kangming Biotech, Shuzhou, China).

### Detection of Free PAs in Plant Materials

Sample extraction was performed as previously described by Chen et al. (2018), with some modifications. Given the interference of various metabolites in roots and leaves of tea, the freeze-dried extracts were applied to UPLC-QTOF/MS for content detection. The Agilent 1290 Infinity II series (Agilent Tech., Santa Clara, USA) ultra-high performance liquid chromatograph (UHPLC) was used to separate the target compounds through a Waters ACQUITY UPLC BEH Amide column (100 × 2.1 mm, 1.7 μm, Waters, Milford, USA). The mobile phase consisted of 1% formic acid in water (A) and 1% formic acid in acetonitrile (B). The elution gradient was as follows: 0 min, 5% A; 1 min, 25% A; 4 min, 30% A; 5–8 min, 55% A; 8.5–13 min, 25% A. The oven temperature was set at 40°C, the sample tray was set at 4°C, and the injection volume was 1 μl. Agilent 6460 triple quadrupole mass spectrometer (QMS, Agilent Tech., Santa Clara, USA) equipped with an AJS-ESI ion source was used for mass spectrometry analysis in multiple reactions monitoring (MRM) mode. The electrospray ionization (ESI) source conditions were set as follows: Capillary voltage 4000 V or -3500 V, nozzle voltage 500 V or -500 V, gas (N_2_) temperature 300°C and flow rate 5 L/min, sheath gas (N_2_) temperature 250°C and flow rate 11 L/min, nebulizer 45 psi. All mass spectral data acquisition and quantitative analysis of target compounds were performed by Agilent Mass Hunter Work Station Software (B.08.00, Agilent Technologies).

### Data Source and Statistical Analysis

One-way analysis of variance (ANOVA) was performed on the experimental data using SPSS 22, and multiple comparisons were performed using Duncan to analyze the significance of the difference between the two treatments at the 0.05 level.

## Results and Analysis

### Screening and Identification of CsPAO Genes

Using the *AtPAO* and *OsPAO* sequences as queries, seven *PAO* genes were identified from the *Camellia sinensis* genome database (TPIA http://tpia.teaplant.org/), and named *CsPAO1-7*. The length of the *CsPAO* ORF ranged from 1326 bp (*CsPAO5*) to 2385 bp (*CsPAO3*). The predicted MV range was 48.53 kDa (*CsPAO5*) to 87.11 kDa (*CsPAO3*), and the theoretical isoelectric point (pI) range was 5.03 (*CsPAO6*) to 7.28 (*CsPAO7*), which suggested that the polypeptides encoded by these genes had notable differences. Other detailed information on these genes, including gene position and subcellular localization prediction, are listed in **Table 1**.

**Table 1:** Polyamine oxidase genes in *Camellia sinensis*.

Gene name	Position^a^	Nucleotide length	CDS	Intron^b^	Protein (aa)	MV(kDa)	pI	Subcellular localization	Unigene	Gene ID^c^
*CsPAO1*	Scaffold3487:197709-199394-	1685	1686	0	561	61.89	5.89	Cytoplasm	CssPBTrans054340	MT316116
*CsPAO2*	Scaffold191:515007-516665-	1658	1659	0	552	60.85	5.68	Cytoplasm	CssPBTrans054340	MT316120
*CsPAO3*	Scaffold1374:981112-983496+	2384	2385	0	794	87.11	6.38	Chloroplast	CssPBTrans049356	MT316119
*CsPAO4*	Scaffold864:1704589-1710385-	5796	1458	9	485	53.83	5.41	nucleus	CssPBTrans054870	MT316117
*CsPAO5*	Scaffold5186:292427-301668+	9241	1326	7	441	48.53	6.73	nucleus	CssPBTrans019845	MT316118
*CsPAO6*	Scaffold10863:176411-193004+	16593	1338	8	445	48.29	5.03	Vacuolar	CssPBTrans019845	MT316114
*CsPAO7*	Scaffold671:734291-737947+	3656	2274	1	757	83.78	7.28	Chloroplast	CssPBTrans049356	MT316115

a Physical position on tea plant WGS (TPIA, http://tpia.teaplant.org/).

a The number of intron in coding region.

c The accession numbers are from NCBI (https://www.ncbi.nlm.nih.gov/).

### Systematic Evolution, Gene Structure, and Tissue-Specific Expression of CsPAOs

**Figure 1:**
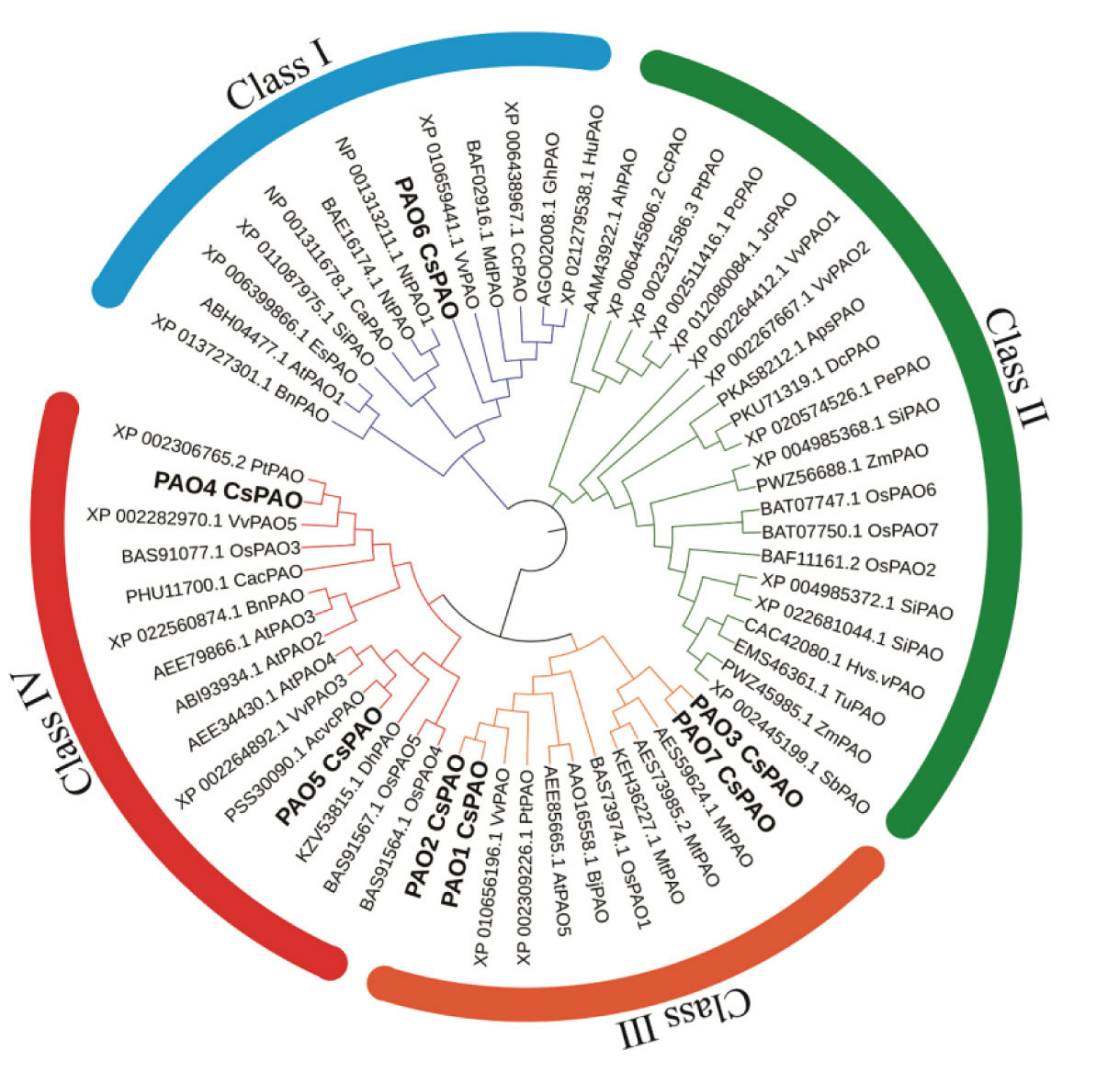
Phylogenetic analysis of *CsPAO* genes. The colored branch indicates the total PAO proteins classed into four groups, and seven CsPAO proteins classed into three groups according the amino acid sequence alignment by MEGA 7.0 and ITOL.

To identify the candidate PAO proteins in tea plant, PAOs of *Arabidopsis*, *Oryza sativa* and *Zea may*, were collected to construct a phylogenetic tree by MEGA 7.0 (**Figure 1**). The alignment of the tea PAO amino acid sequences with that of the other plants showed that all PAO proteins were divided into four subfamilies, and the *CsPAOs* were distributed in three groups, clade I, III, and IV. No gene sequence belonging to clade II has been selected from the TPIA database. Among them, *CsPAO6* belongs to clade I, *CsPAO1-3* and *CsPAO7* belong to clade III, and *CsPAO4*-5 belong to clade IV. According to the functional classification of the rice *OsPAO* family (Sagor et al., 2017), PAOs in clade II mainly acted on the TC-reaction of PAs, while clades I, III, and IV acted to catalyze the BC-reaction of Spm to Spd. Amino acid sequence alignment results showed that the CsPAO family was highly unified (**Supplemental Figure 1**), and all family members had typical amino oxidase conserved domains. Meanwhile, some PAO sequences with unique SWIRM domains have also been screened in rice, tomato and other plants, whose main function is to catalyze the demethylation of H3K4 histone lysine, which belongs to the clade of histone lysine-specific demethylases, rather than true PAO. In this study, sequences of similar structure were not identified.

**Figure 2:**
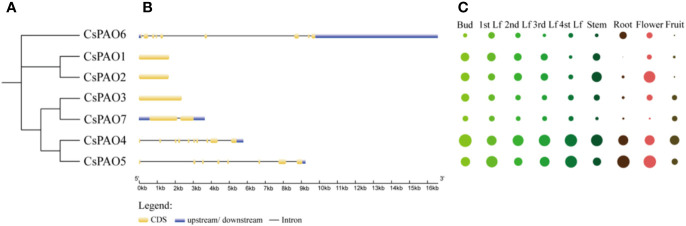
Characteristics description of *CsPAO* gene family members, including phylogenetic tree **(A)**, genetic structure analysis **(B)**, and tissue specific expression **(C)**. Different colors represent different tissues and organs, and the circle size represents relative expression level.

Phylogenetic analyses of tea *PAO* genes were performed (**Figure 2A**), and corresponding genetic structure analyses by GSDS website were used to reveal the exon and intron structures of *CsPAOs* (**Figure 2B**). The *CsPAO1*, *CsPAO2*, and *CsPAO3* genes did not have any introns in their genomic sequences. *CsPAO7* had one intron, *CsPAO5* had seven introns, while *CsPAO4* and *CsPAO6* had nine introns. These differences indicated that *CsPAOs* are complex in evolution, and have different expression regulation modes.

The tissue-specific expression of *CsPAOs* in the bud, first leaf, second leaf, third leaf, fourth leaf, root, stem, flower and fruit were detected by qRT-PCR (**Figure 2C**). Seven *CsPAO* genes were expressed in all tissues. The relative expression levels of *CsPAO4* and *CsPAO5* were higher in all tissues, *CsPAO4* was the highest in bud, and *CsPAO5* was the highest in root and flower among all *CsPAO* genes. The expression levels of *CsPAO6* and *CsPAO7* were relatively low in all tissues, while *CsPAO1* was not detected in root.

### Cloning and Heterologous Expression of Partial CsPAO Genes

**Figure 3:**
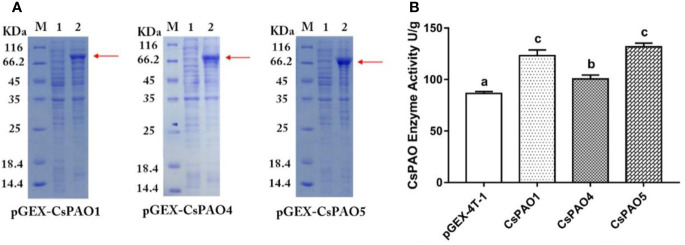
Result of heterologous expression of partial CsPAO proteins. **(A)** SDS-PAGE analysis the prokaryotic expression result of pGEX-CsPAO1, pGEX-CsPAO4, and pGEX-CsPAO5 fusion protein, respectively. M, protein marker; 1, preinducted supernatant; 2, induced supernatant. **(B)** Results of PAO enzyme activity of fusion proteins.

The identified *CsPAO1*, *CsPAO4*, and *CsPAO5* full-length gene sequences were cloned and ligated into the pGEX-4T-2 vector for heterologous expression. SDS-PAGE results showed that CsPAO1, CsPAO4, and CsPAO5 expressed corresponding fusion proteins (**Figure 3A**). The expressed fusion protein was purified by GST column and assayed for enzyme activity. pGEX-4T-2 was used as an empty vector control to detect the enzyme activity of the three PAOs in tea plants. The results showed that three recombinant *E. coli* strains had the catalytic activity of PAO (**Figure 3B**).

### Effects of Abiotic Stress on CsPAO Gene Expression Patterns

**Figure 4:**
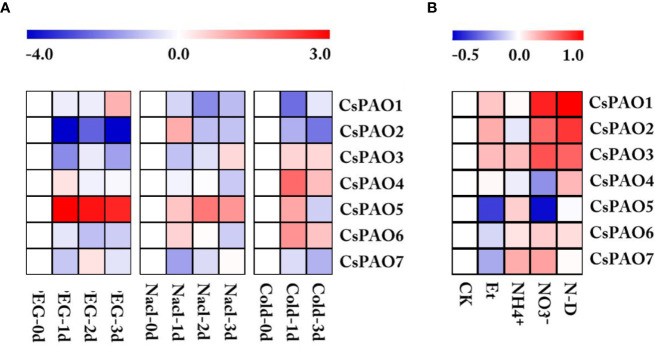
Heatmap of *CsPAO* genes expression under different abiotic stress and nitrogen nutrition treatment. **(A)** Stress treatment including drought (PEG), salt (Nacl) and cold stress. **(B)** Nitrogen nutrition treatment including nitrogen-deficiency (N-D), nitrate nitrogen (NO_3_^-^), ammonium nitrogen (NH_4_^+^), ethylamine (Et), and normal nitrogen (CK). RPKM value was determined by RNA-seq data and standardized by log2 conversion.The color scale represents the gene expression level, blue represents low expression, and red represents high expression.

Based on the analysis of *cis*-elements in the promoter regions, we screened the *CsPAO* genes transcriptome data of tea leaf from TPIA under different stress treatments, including drought, salt, and cold. After homogenizing the RPKM values of corresponding unigenes, a heat map was drawn for further analysis (**Figure 4**). The *CsPAO* genes in tea leaves showed different response strategies to different abiotic stresses. *CsPAO5* expression was significantly up-regulated in response to drought, salt and cold stresses. The expression of *CsPAO2* was down-regulated, especially in drought stress. The response of tea leaves to cold stress was very quick, and the expression changes were most obvious within 24 h. *CsPAO3*, *CsPAO4*, and *CsPAO6* showed up-regulated expression, while *CsPAO1* and *CsPAO7* showed down-regulated expression in cold stress.

We also analyzed the response of tea *CsPAO* gene family to nitrogen nutrition stress using the transcriptome data (unpublished data) from our laboratory. The results showed that *CsPAO1*, *CsPAO2*, and *CsPAO3* were more sensitive to nitrogen nutrition, and showed different degrees of up-regulated expression in nitrogen deficiency, nitrate nitrogen and ethylamine treatments. *CsPAO5* and *CsPAO7* did not respond to nitrogen deficiency. *CsPAO5* was significantly down-regulated under nitrate nitrogen and ethylamine treatments, but was up-regulated under ammonium nitrogen treatment. Therefore, its internal regulatory mechanism needs further investigation.

### Effects of Exogenous ABA on CsPAO Gene Expression and PAs Content

**Figure 5:**
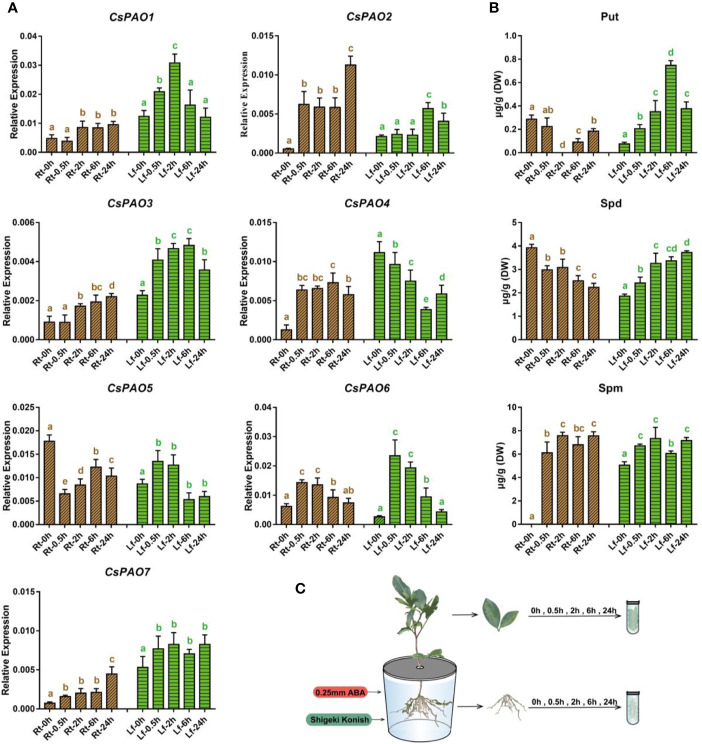
Effects of exogenous ABA treatment on polyamine metabolism in tea seedlings. **(A)** qRT-PCR analysis the change of relative expression of *CsPAO* genes. **(B)** HPLC detected the polyamines contentin different organs of tea seedlings. **(C)** Schematic diagram of hydroponic tea seedlings and sampling. Sampling the fibrous roots (Rt) and young leaves (Lf) of tea seedlings at 5 time points (0, 0.5, 2, 6, 24 h) after 0.25 mM ABA treatment. The s.d. of three biological replicates is shown using error bar. Three technical replicates were performed and each gave the similar result. Different letters (a, b, c) indicate statistical significance among treatments using one-way analysis of variance (ANOVA) test and a Fisher’s least significant difference (LSD) at the 5% significance level.

Given the presence of ABRE *cis*-elements in multiple *CsPAO* gene promoter regions and related reports of ABA-regulated PA metabolism in other plants, exogenous ABA was used to treat tea seedlings to explore its regulatory effect on *CsPAO* genes in different tissues. In order to ensure treatment consistency, we added the same content of ABA to the hydroponic solution, and then selected the same growth tea seedlings and transferred them to the hydroponic tank (**Figure 5C**). The results showed that ABA signal transduction was very rapid and was not significantly affected from roots to leaves.

The qRT-PCR results (**Figure 5A**) showed that except for *CsPAO5*, which was significantly down-regulated, the other six *CsPAOs* were up-regulated in the roots. Among them, *CsPAO2* showed the most significant response. The expression of *CsPAO6* gradually reverted to normal level after 6 h of treatment, while the remaining genes maintained a high response, and *CsPAO2* and *CsPAO7* remained significantly increased after 24 h of ABA treatment. In the leaves, except for the decreased expression of *CsPAO4*, other *CsPAO* genes showed an initial increase and subsequent decrease. Among them, *CsPAO6* showed the fastest response, with the highest expression level at 30 min after treatment, and most genes reverted to the baseline after 24 h.

The content of PAs in different tissues of tea plants treated with ABA was detected by LC-MS (**Figure 5B**), and it was found that Put metabolism had a greater influence, and the metabolism changes of root and leaf showed completely opposite trends. The ABA treatment resulted in a significant decrease in Put content in roots. After 2 h, Put content was nearly undetectable, but gradually recovered. In contrast, Put content increased significantly in the leaves and peaked after 6 h of treatment, increased nearly 7 times, and then gradually dropped. The change of Spd was similar to that of Put, showing an ascending tendency in leaves and descending tendency in roots, but the changes were relatively stable. Spm was detected in the tea samples treated with ABA, the content change of Spm in roots and leaves was consistent, and both increased slowly. The above results showed that exogenous ABA activated the expression of most *CsPAO* genes, promoted the production of Spd by Put, and subsequently generated Spm. Thereafter, Spd and Spm were decomposed under the action of CsPAOs.

#### 3.6 Effects of Anaerobic Treatment on CsPAO Gene Expression and PAs Content in Tea Plants

**Figure 6:**
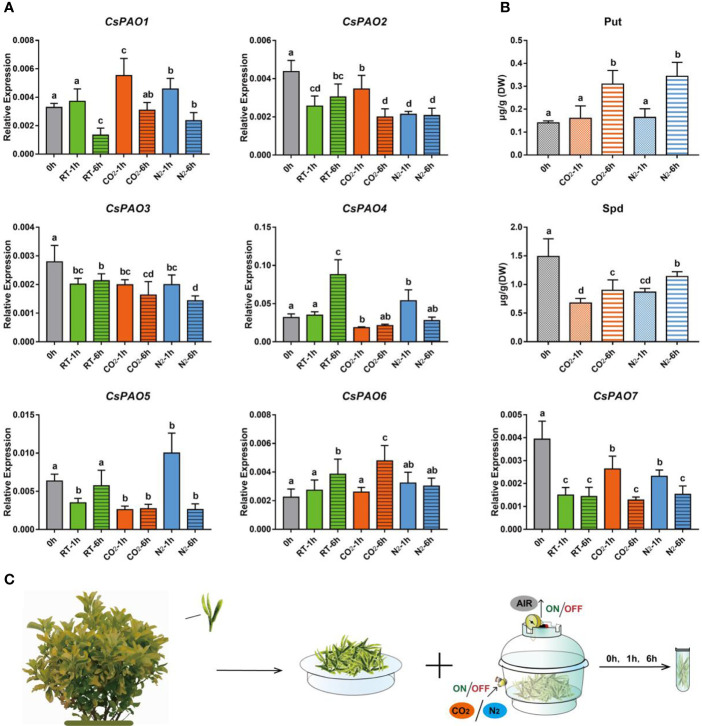
Effects of anaerobic treatment on polyamine metabolism in tea plant. **(A)** qRT-PCR analysis the change of relative expression of *CsPAO* genes. **(B)** HPLC detected the polyamines contentin in new shoots of tea plant after different anaerobic treatment (N_2_ or CO_2_). **(C)** Schematic diagram of sampling and anaerobic treatment, gray bars indicate the initial samples, green bars indicate natural spreading treatment (RT), orange bars indicate filled-CO_2_ treatment, and blue bars indicatefilled-N_2_ treatment. The s.d. of three biological replicates is shown using error bar. Three technical replicates were performed and each gave the similar result. Different letters (a, b, c) indicate statistical significance among treatments using one-way ANOVA test and a Fisher’s LSD at the 5% significance level.

Anaerobic treatment is often used to increase the content of gamma-aminobutyric acid (GABA) in tea leaves to produce Gabaron tea with more health benefits, and PA metabolism is a main pathway for GABA synthesis. We conducted different anaerobic treatments (**Figure 6C**) on picked tea leaves to explore their effects on *CsPAO* expression and PA metabolism in tea. The qRT-PCR results showed that the expression of *CsPAO* genes presented a differential response under anaerobic treatment *in vitro*. *CsPAO2*, *CsPAO3*, and *CsPAO7* showed down-regulation compared with fresh leaves. *CsPAO1* and *CsPAO6* were significantly up-regulated under filled-CO_2_ treatment, while *CsPAO4* and *CsPAO5* were significantly up-regulated under filled-N_2_ treatment. The expression of *CsPAO6* was up-regulated under each treatment, which was speculated to be related to the presence of multiple ARE elements in its promoter region (in response to anaerobic induction) (**Figure 6A**).

Two PAs, Put and Spd, were detected in picked tea leaves by LC-MS. The Put content increased significantly after 6 h of anaerobic treatment, while the Spd content decreased significantly under anaerobic treatment, but slightly increased after 6 h of anaerobic treatment. Spm was detected in fresh tea leaves, but not after anaerobic treatment (**Figure 6B**).

## Discussion

PAs act as signaling molecules in plants, in addition to regulating growth and protection. However, the mechanism of PA metabolism and regulation, as well as PAs signal transduction pathway in tea plants are not well understood. The purpose of this study was to reveal how *CsPAOs* respond to various abiotic stresses and regulate the process of PA metabolism, in order to screen key *CsPAO* genes that are more sensitive to stress and provide evidence for further study on the signal transduction pathway and catabolism of PAs.

### Gene Structure of CsPAOs

*PAO* gene families have been reported in A*rabidopsis thaliana*, rice, citrus, cotton, and tomato (Liu et al., 2014a; Kusano et al., 2015; Wang and Liu, 2015; Cheng et al., 2017; Hao et al., 2018). Herein, we discussed the possible role of *CsPAOs* based on the classification of their members in phylogenetic tree and their expression patterns under different stresses. Phylogenetic relationship analysis was conducted on seven *CsPAO* genes, and compared with the genetic structure in other plants, they were classified as clade I, clade III and clade IV, without clade II (**Figure 1**), which was consistent with the results of *PAO* genes in tomatoes (Hao et al., 2018). *CsPAO6* is different from *AtPAO1* and other clade I members (Tavladoraki et al., 2006), and is localized on vacuoles, indicating that it might be involved in the regulation of osmotic pressure. Therefore, *CsPAO6* is speculated to possess the characteristics of clade I members, and can catalyze the BC-reaction of PAs. Four tea genes (*CsPAO1-3*, and *CsPAO7*) have no introns or only one intron (**Figure 3B**), which is similar to *AtPAO5* and *OSPAO1* (Kim et al., 2014; Liu et al., 2014b). Hence, they were classified as clade III. Such members usually act as BC-type enzyme of the PA degradation pathway and localize to cytoplasm or chloroplast. Clade IV members are also PA BC-reaction type, but are localized in peroxisomes (Fincato et al., 2010; Takahashi et al., 2018). *CsPAO4* and *CsPAO5* belonged to this clade, which suggested that they played similar roles as other clade IV members. The clade II members, including barley *HvPAO1*, maize *ZmPAO1*, and rice *OsPAO6* and *OsPAO7*, have the ability to catalyze TC-reactions and are localized in vacuole or apoplast (Cervelli et al., 2000; Cervelli et al., 2006; Sagor et al., 2017; Liu et al., 2014c). No such genes were found in the tea plant genome. However, Wang et al. showed that the *CsPAO4* gene of sweet orange was classified to a clade with *AtPAO5* and *OsPAO1*, and had the ability of TC-reaction (Wang and Liu, 2016), indicating that the function of *PAO* genes in tea needs further study.

### Effects of Exogenous ABA on CsPAO Expression and PA Metabolism

Abscisic acid (ABA), as an important product of plant stress tolerance, and functions as a signal molecule in plant stress response (Cutler et al., 2010). Studies have confirmed that ABA can promote the production of H_2_O_2_ to regulate calcium signals and affect various ABA responses, including stomatal closure (Konstantinos et al., 2010; Hua et al., 2012). PAO-mediated decomposition of PAs produces a large amount of H_2_O_2_, suggesting that ABA regulates PA metabolism in response to environmental stress through a specific signaling cascade (Toumi et al., 2010). These studies have mainly focused on model plants and herbaceous plants, such as *Arabidopsis thaliana* and rice, while studies on perennial woody plants have been very limited. Hence, this study investigated the role of *CsPAO* genes in ABA stress responses and PA catabolism.

Three PAs, putrescine (Put), spermine (Spm), and spermidine (Spd) were detected. Combined with determination of the changes of PAs, ABA was found to have a complex influence on PA metabolism in tea plants, and different regulation of ABA stress tolerance were presented in tea roots and leaves. ABA treatment caused a rapid increase in Put content in leaves, which gradually dropped after 24 h, suggesting that the leaves of tea seedlings could regulate the decomposition of Arg and Org to enhance Put synthesis to cope with the injury of tea leaves caused by ABA (Quinet et al., 2010, Kasinathan and Wingler, 2004). In contrast, the Put content decreased rapidly in the tea roots, was almost untraceable after 2 h, and then gradually increased to the baseline level. It was speculated that the sharp decline in Put content may be caused by two factors, Put was transported from roots to leaves to cope with ABA stress (Fujita and Shinozaki, 2015), and it was also rapidly decomposed under the action of diamine oxidase (DAO) to generate H_2_O_2_ to regulate the Ca^2+^ level of cytosol, while promoting GABA synthesis to alleviate proton stress in the cytoplasm (Zarei et al., 2015). Similarly, in Vicia faba, ABA induced an apoplastic CuAO (DAO) activity as a source of H_2_O_2_ to increase cytosolic Ca^2+^ level to mediate stomata closure (An et al., 2008). Consistent with these observations, the peroxisomal *AtDAO* was shown to be expressed in guard cells in response to the ABA-mediated control of stomata opening (Qu et al., 2014; Ghuge et al., 2015).

Spm was extensively generated in the roots of tea plants after treatment with ABA, and was also slightly increased in the leaves, confirming its role as an indicator of stress tolerance in plants (Ebeed et al., 2017). ABA could promote methionine (Met) to synthesize a large amount of Spm *via* decarboxylated S-adenosylmethionine (dcSAM), and the decomposition of Spm was not obvious at this time. As an intermediate of PA metabolism, Spd exhibited complicated changes, including the synthesis of dcSAM catalyzed by spermidine synthase (SPDS) and the decomposition of PAO. The expression of the *CsPAO* gene family showed significant changes after ABA treatment, similar to abiotic stress, with obvious spatiotemporal specificity. Combined with the expression changes of *CsPAO* gene family, we speculated that *CsPAO4* plays an important role. After ABA treatment, the expression of *CsPAO4* was significantly up-regulated in the roots, while the content of Spd was decreased, indicating that Spd in the roots was mainly catabolic at this time. *CsPAO4* perhaps plays a role of terminal catabolism, which was similar to the *PAO* genes identified in corn (*ZmPAO1*), rice (*OsPAO7*), and sweet orange (*CsPAO4*) involved in TC reaction of PAs (Cervelli et al., 2001; Liu et al., 2014c; Wang and Liu, 2016). Meanwhile, the expression of *CsPAO4* was significantly down-regulated in the leaves, while the content of Spd was increased, indicating that the TC-reaction of CsPAO4 was significantly inhibited. The expression of other *CsPAO* genes was significantly up-regulated at this time, which may be consistent with the role of PAO in *Vitis vinifera* and *Arabidopsis* (Hou et al., 2013), mainly involved in the BC-reaction. These *CsPAO* genes were used to promote the generation of Spd and Put to cope with ABA stress in leaves. However, the function of *CsPAO4* needs further study.

### Effects of Anaerobic Treatment on CsPAO Expression and PA Metabolism

Some waterlogged plants showed elevated levels of plant hormones, including ABA, zeatin riboside (ZR), and ethylene. Occasionally, plants showed similar regulatory effects on ABA and osmotic stress, when subjected to drought stress or root tip growth (Hatmi et al., 2014; Hatmi et al., 2018). However, during tea processing, special anaerobic methods are often used to improve osmotic stress to obtain functional tea with high GABA content (Lin et al., 2012). In this process, the respiration of post-harvest tea leaves was inhibited by complete anaerobic or inert gas anaerobic treatment, thereby activating CaM to increase the enzyme activity of glutamic acid decarboxylase (GAD) and promoting GABA synthesis (Wu et al., 2017; Chen et al., 2018). Given that the PA metabolism pathway is also an important branch of GABA synthesis (Seiler, 2004), this study explored the response mechanism of the *CsPAO* gene family to anaerobic stress.

Spm was nearly undetectable in leaves *in vitro* during anaerobic treatment, and the content of Spd also decreased obviously, which was inconsistent with the typical PA accumulation induced by osmotic stress (Ebeed et al., 2017). These results indicated that PAs showed obvious oxidative decomposition in picked tea leaves under anaerobic treatment, which was similar to the performance of plants when infected by certain pathogens (Saloua et al., 2013; Saloua et al., 2018). Combined with the results of promoter analysis, multiple ARE *cis*-elements were identified upstream of the *CsPAO6* promoter. Additionally, qRT-PCR results showed that *CsPAO6* expression was up-regulated under different anaerobic treatments, suggesting that it might be involved in the TC-reaction of PAs. Although the anaerobic treatment of tea leaves was often accompanied by an increase in endogenous ABA, the results of anaerobic treatment were significantly different from ABA treatment. The expression levels of *CsPAO2*, *CsPAO3* and *CsPAO7* were down-regulated after anaerobic treatment, while *CsPAO1* expression level was initially up-regulated and subsequently down-regulated, indicating that the members of clade III gene subfamilies of BC-type were inhibited. The performances of *CsPAO4* and *CsPAO5* in clade IV were relatively consistent. They were down-regulated under filled-CO_2_ treatment, but initially up-regulated and subsequently down-regulated under filled-N_2_ treatment. Tea plant has complex coping strategies to different types of anaerobic stress (Qi et al., 2018), which also affected the catabolic process of PAs, so the BC-reaction of PAs was generally inhibited by anaerobic stress.

**Figure 7:**
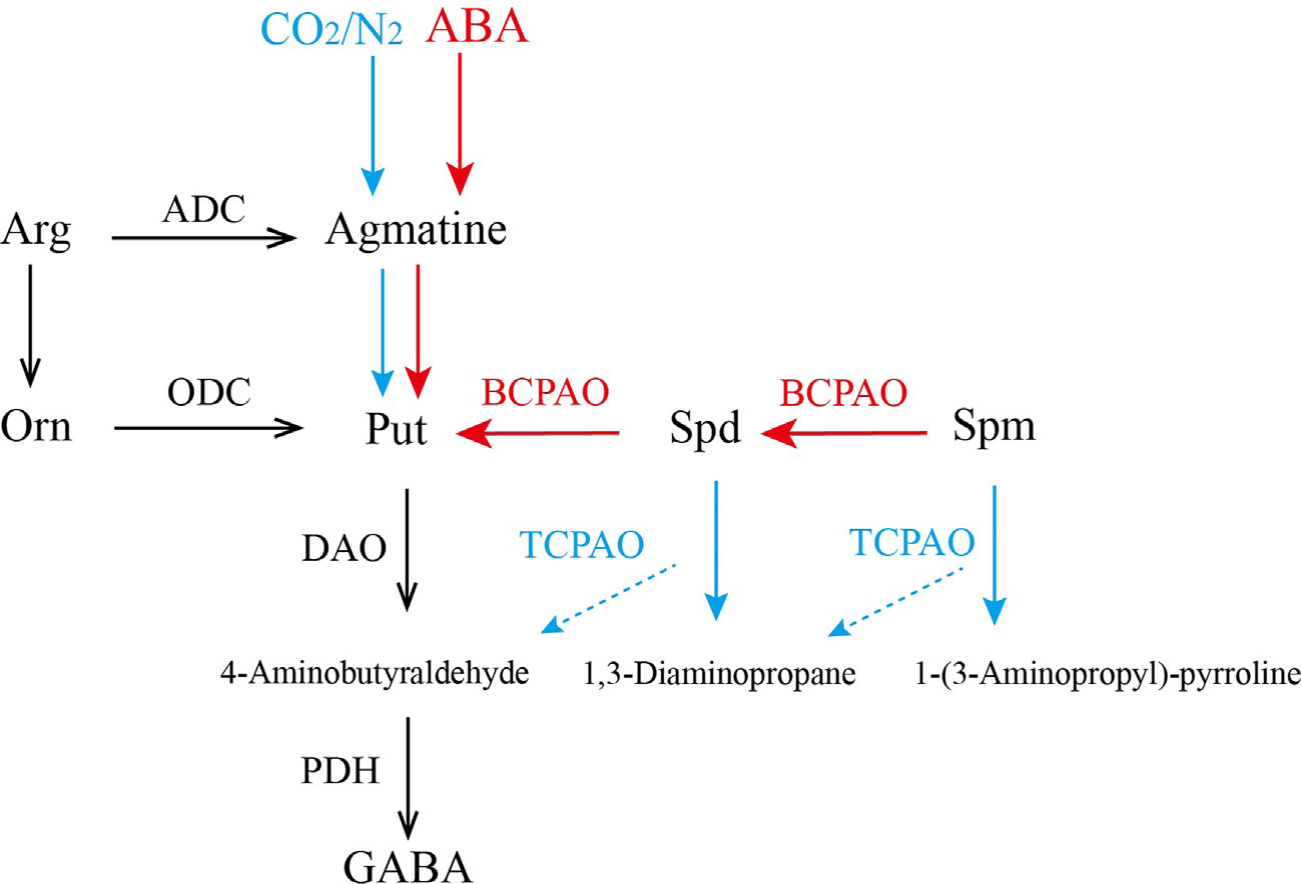
The process of polyamines metabolism under anaerobic treatment and ABA treatment. Red arrow indicates that the main function of CsPAOs under ABA treatment is BC-type, while blue arrow indicates that the main function of CsPAOs under anaerobic treatment is TC-type.

The above results are summarized in **Figure 7**. The application of exogenous ABA promoted the synthesis of PAs and activated the expression of *CsPAO* genes of BC-type (red arrow), resulting in a significant increase of some PAs. However, under anaerobic treatment, most *CsPAOs* involved in the BC pathway were inhibited, the content of PAs decreased, and the content of GABA increased. At this time, TC-type of *CsPAO* was dominant in tea leaves (blue arrow). Combining the changes of *CsPAO* gene expression levels and PA contents, and the results of information bioanalysis, we speculated that *CsPAO1, CsPAO3*, and *CsPAO5* participate in the BC pathway; while *CsPAO4* and *CsPAO6* participate in the TC pathway. The exact functions of the PAO gene family members need to be verified by more elaborate and systematic experiments.

## Data Availability Statement

The data generated for this study can be found in NCBI. The accession number of the cold-processed RNA-Seq dataset is SRA061043. The accession number for the PEG and salt-treated. RNA-Seq dataset is PRJEB11522.

## Author Contributions

MSL, JL, and MT performed the research. MRL analyzed the data. YH and E-HX contributed the bioinformatics analysis/computational tools. XW and QC participated in the research design. MSL and QC wrote the paper. All authors contributed to the article and approved the submitted version.

## Funding

The authors appreciate the funding support from the National Natural Science Foundation of China (No. 31500566), the College Excellent Youth Talent Support Program of Anhui Province (gxyqZD2019014), and the Agricultural Research System of China (CARS-19).

## Conflict of Interest

The authors declare that the research was conducted in the absence of any commercial or financial relationships that could be construed as a potential conflict of interest.
