# Supplementary material for: Genome-Wide Identification of Seven Polyamine Oxidase Genes in Camellia sinensis (L.) and Their Expression Patterns Under Various Abiotic Stresses
Source: Front Plant Sci. 2020 Sep 4;11:544933. doi: 10.3389/fpls.2020.544933 (PMC7500180; doi:10.3389/fpls.2020.544933)
Supplement: Supplementary file 1 [file Table_1.docx]

**Supplement Table 1.**

**Detail of primer sequences used for analyzing the expression of *CsPAO* genes in tea plant by quantitative real time PCR.**

| **Gene name** | **Primer name** | **Primer sequence 5’-3’** | **Length** | **Start** | **Product Size (bp)** |
| --- | --- | --- | --- | --- | --- |
| *CsPAO1* | *CsPAO1*-F | GACTCGAATTGGTGGAAGGA | 20 | 126 | 170 |
|  | *CsPAO1*-R | TCATCCAAAAACCCATCCAT | 20 | 296 |  |
| *CsPAO2* | *CsPAO2-*F | TTTGATCCACCACTTCCACA | 20 | 958 | 161 |
|  | *CsPAO2-*R | GGAGTCTGACCGCTGAAAAG | 20 | 1119 |  |
| *CsPAO3* | *CsPAO3*-F | GGGGTGGGGAGATTGATACT | 20 | 1562 | 162 |
|  | *CsPAO3*-R | TCCACTGGGGACATCTTTTC | 20 | 1724 |  |
| *CsPAO4* | *CsPAO4-*F | TTGCTGCAGATGCTGATACC | 20 | 593 | 201 |
|  | *CsPAO4*-R | GATCGCCCATCTTCAACTGT | 20 | 794 |  |
| *CsPAO5* | *CsPAO5*-F | GGAAAGCCCGAAGATGTGTA | 20 | 1234 | 158 |
|  | *CsPAO5*-R | CCTCTCCATGAGATGCCTTC | 20 | 1392 |  |
| *CsPAO6* | *CsPAO6*-F | ATTGGTGTTCTGCAAAGCGAC | 21 | 739 | 197 |
|  | *CsPAO6*-R | TCAGGTATGTCAGGCCCAAAC | 21 | 935 |  |
| *CsPAO7* | *CsPAO7*-F | TCAGCGAATCCATTGCACTCT | 21 | 1009 | 237 |
|  | *CsPAO7*-R | ACCCATCTTCACTTTCCCGAA | 21 | 1245 |  |

**Supplement Table 2.**

**Detail of full-length primer sequences of some *CsPAO* genes in tea plant by whole-length clone.**

| **Gene name** | **Primer name** | **Primer sequence 5’-3’** |
| --- | --- | --- |
| *CsPAO1* | *CsPAO1*-pGEX-4T-2-*Nco Ⅰ-F* | AATCCATGGATGGTGGCCAAGAAG |
|  | *CsPAO1*-pGEX-4T-2- *Hind Ⅲ-R* | ACGAAGCTTTCATACCCCATTGCAA |
| *CsPAO4* | *CsPAO4*-pGEX-4T-2-*Nco Ⅰ-F* | TAACCATGGATGGAGTCGCGAGAAC |
|  | *CsPAO4*-pGEX-4T-2- *Hind Ⅲ-R* | CGCAAGCTTTTACATACGAGAGATCAAC |
| *CsPAO5* | *CsPAO5*-pGEX-4T-2-*Nco Ⅰ-F* | GGGCCATGGATGGATCTCAAAGACTC |
|  | *CsPAO5*-pGEX-4T-2- *BamHl-R* | TCGGATCCTCAAATCCTTGAGATCTGAAG |

**Supplement Table 3.**

**Composition of standard culture solution of Shigeki Konish**

| **Element** | **Compound** | **Concentration(mg/L)** |
| --- | --- | --- |
| N-NH4^+^ | (NH_4_)_2_SO_4_ | 30 |
| N-NO3^-^ | Ca(NO_3_)_2_·4H_2_O | 10 |
| P | KH_2_PO_4_ | 3.1 |
| K | KH_2_PO_4_、K_2_SO_4_ | 40 |
| Ca | CaCl_2_·2H_2_O | 20 |
| Mg | MgSO_4_·7H_2_O | 25 |
| Fe | Fe-EDTA | 0.35 |
| B | H_3_BO_3_ | 0.1 |
| Mn | MnSO_4_·4H_2_O | 1 |
| Zn | ZnSO_4_·7H_2_O | 0.1 |
| Cu | CuSO_4_·5H_2_O | 0.025 |
| Mo | Na_2_MoO_4_·2H_2_O | 0.05 |
| Al | Al_2_(SO_4_)_3_·16-18 H_2_O | 10 |

**Supplement Figure 1**


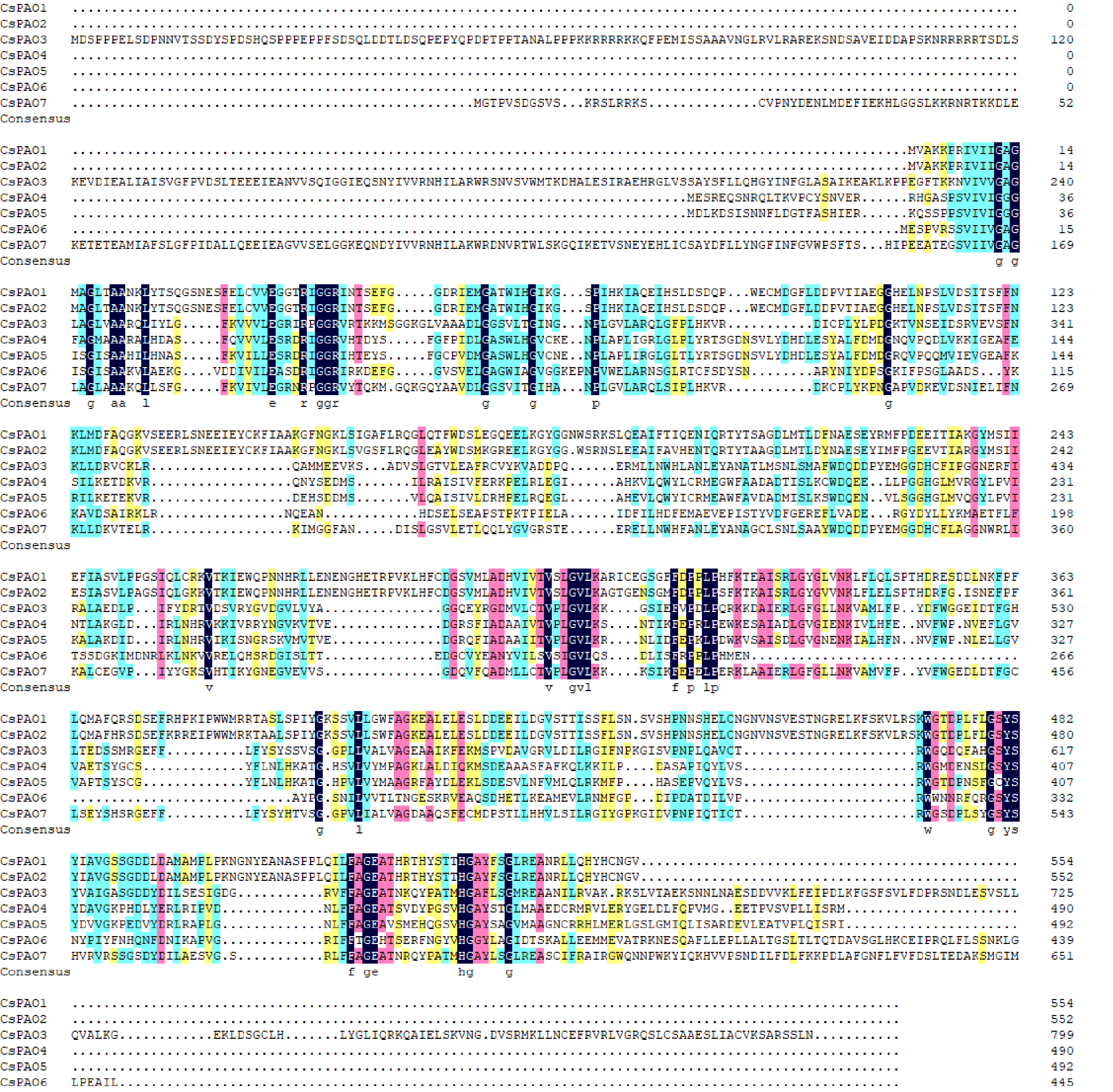


Fig. 1. Alignment analysis the peptide sequences of *CsPAO* genes.
